# Efficacy of biofeedback rehabilitation based on visual evoked potentials analysis in patients with advanced age-related macular degeneration

**DOI:** 10.1038/s41598-020-78076-w

**Published:** 2020-11-30

**Authors:** Tommaso Verdina, Stefania Piaggi, Vanessa Ferraro, Valeria Russolillo, Riccardo Peschiera, Johanna Chester, Rodolfo Mastropasqua, Gian Maria Cavallini

**Affiliations:** 1 Institute of Ophthalmology, University of Modena and Reggio Emilia, Modena, Italy; 2 Department of Dermatology, University of Modena and Reggio Emilia, Modena, Italy

**Keywords:** Neuroscience, Diseases, Health care, Medical research

## Abstract

Age-related macular degeneration (AMD) is a progressive and degenerative disorder of the macula. In advanced stages, it is characterized by the formation of areas of geographic atrophy or fibrous scars in the central macula, which determines irreversible loss of central vision. These patients can benefit from visual rehabilitation programmes with acoustic “biofeedback” mechanisms that can instruct the patient to move fixation from the central degenerated macular area to an adjacent healthy area, with a reorganization of the primary visual cortex. In this prospective, comparative, non-randomized study we evaluated the efficacy of visual rehabilitation with an innovative acoustic biofeedback training system based on visual evoked potentials (VEP) real-time examination (Retimax Vision Trainer, CSO, Florence), in a series of patients with advanced AMD compared to a control group. Patients undergoing training were subjected to ten consecutive visual training sessions of 10 min each, performed twice a week. Patients in the control group did not receive any training. VEP biofeedback rehabilitation seems to improve visual acuity, reading performances, contrast sensitivity, retinal fixation and sensitivity and quality of life in AMD patients.

## Introduction

Age-related macular degeneration (AMD) is a progressive and degenerative disorder of the macula, representing the leading cause of severe visual loss and legal blindness among elderly (> 65 years) in the western population^1^. AMD leads to a progressive and irreversible loss of central vision, due to both the formation of areas of geographic atrophy and fibrous scars in the central macula, thus causing a central scotoma, which determines visual impairment^2,3^.

Despite recent research advancements for innovative retinal therapies, including gene and cellular therapies, there are currently no effective treatments for advanced stages of AMD and the prognosis of this disabling disease is poor.

Due to central macular atrophy, patients with low central vision attempt to develop an eccentric fixation area, also known as Preferred Retinal Location (PRL). The PRL is generally located in a healthy part of the retina, often on the superior or horizontal meridian of the fovea^4–6^. It has been shown that patients with central vision loss can develop multiple PRLs, located in different areas depending upon different tasks^7–10^. However, patients are often unaware of how to use the PRLs and where exactly to move the eye in order to fixate objects in everyday skills.

Recently, the use of support for neuronal plasticity of the visual system in adults has grown. Visual rehabilitation of healthy eccentric areas in patients with macular diseases with loss of central fixation, with specific training for the reorganization of the primary visual cortex, has proven advantageous^11–14^. Rehabilitative programs with acoustic “biofeedback” mechanisms instruct the patient to move fixation from the central degenerated macular area to the adjacent healthy PRL^15–17^. Biofeedback rehabilitation programs can be performed with microperimetry or with visual evoked potentials (VEP) real-time analysis.

Retimax (CSO, Firenze, Italy) is a medical device for the detection, recording, amplification and visualization of ocular electrical conduction. It combines electrophysiological measurements with rehabilitative intervention, especially useful in the context of low vision. The “Vision Trainer” module is specifically designed for visual rehabilitation, and it includes an auditory biofeedback system combined to VEP measurements, which has been shown to be an efficient technique in helping patients with different ocular diseases to increase visual performance^18,19^. The VEP recorded at the level of the visual cortex is digitally analyzed in real-time, with direct patient feedback in the form of an acoustic signal. The signal is related to the amplitude and latency of the VEP, thus to the quality of vision. The more the patient maintains a stable fixation with the healthy paracentral PRL, the greater and more stable the intensity of the sound will be. This rehabilitative system instructs the patient to voluntarily control and coordinate ocular movements in order to use the healthy PRL, increasing visual performances by the stimulation of the visual pathway.

Currently, the VEP biofeedback rehabilitation training program has been evaluated for patients affected by amblyopia^18^ and acquired brain injury^19^, reporting significant improvements in visual function and fixation stability. To our knowledge, there are no current studies available investigating the utility of VEP biofeedback rehabilitation for AMD patients. The authors hypothesize that AMD patients may have neuronal plasticity and be suitable for VEP rehabilitation which should improve quality of life. The current study aims to evaluate the efficacy of VEP biofeedback rehabilitation with Retimax Vision Trainer and quality of life in a group of consecutive patients with advanced stage AMD compared with a control group.

## Methods

This prospective, non-randomized, observational study enrolled a group of consecutive patients with advanced AMD, recruited at the Institute of Ophthalmology, University of Modena (Italy). The Ethical Committee of the University of Modena and Reggio Emilia (Modena, Italy) approved the study (prot. NO. 6684/19, study number 968/2018/DISP/AOUMO). The study was conducted in accordance with the Declaration of Helsinki. Registration number in a public registry was obtained (number NCT04084587). All patients gave their written, informed consent.

Protocol criteria outlined the inclusion of < 90 years old patients with advanced atrophic AMD, unstable or relatively unstable fixation, best corrected visual acuity (BCVA) between 20/100 (0.7 logMar) and 20/320 (1.3 logMar) in the eye with the highest BCVA, and good patient compliance. Criteria specified the exclusion of patients ≥ 90 years old with active neovascular AMD, stable fixation, significant cataract, glaucoma, optic nerve disease/abnormalities, diabetic retinopathy, previous ocular surgery other than cataract intervention and patients unable to collaborate during the Retimax or the microperimetric evaluation.

Patients were assigned to the training or control groups depending upon their availability to attend 2 training sessions/week for the study period of 5 weeks. Patient enrollment and training was performed between May 2018 and May 2019.

All patients underwent a complete ophthalmic examination at baseline (T0) and after 6 weeks from enrolment (T1). Examinations included BCVA, contrast sensitivity (CS), reading ability, vision-related quality of life test (VFQ-25), microperimetry with fixation analysis. BCVA was evaluated with the Early Treatment Diabetic Retinopathy Study (ETDRS) charts (Lighhouse Int., New York, NY) at 4 m and assessment was in letters. CS was evaluated using Pelli-Robson Contrast Sensivity Chart (Precision Vision La Salle Illinois, USA) at a distance of 2 m. Reading ability was evaluated with MNRead charts (Precision Vision La Salle Illinois, USA) distributed in the Italian version by Carl Zeiss (Jena GmbH, Germany) at a 25 cm distance. For all patients, we added a + 3.00 spherical lens. Maximum reading speed (MRS) was measured in words per minute (wpm), reading acuity was assessed in logMAR units. Microperimetry was performed in the eye with the highest BCVA with spectral domain OCT (OCT-SLO, Optos, Scotland, UK), following pupil dilation with 0.5% tropicamide and 2.5% phenylephrine and a period of adaptation of 10 min to dim room illumination. A pattern with 28 locations in the macular area was used to assess sensitivity. “White” test lights (stimulus size Goldmann III, 200 ms in duration) were presented on a dim “white” background (1.27 cd/m2) using a 4–2 procedure. With the untested eye occluded, the patient was asked to maintain fixation on a 2° green point (fixation target). All patients had prior experience of microperimetric tests of visual function. Retinal sensitivity was measured and recorded with the average sum of all test locations obtained during the test. We analyzed location and stability of fixation. Stability was defined in terms of the percentage of fixation points that fell within a 2° and 4° diameter circle during the test defining stable (more than 75% of recorded fixation points within the 2° diameter circle), relatively unstable (less than 75% within the 2° diameter circle but more than 75% within the 4° diameter circle) and unstable fixation (less than 75% fixation points within the 4° diameter circle) as described by Fujii^20^. The National Eye Institute Visual Functioning Questionnaire-25 (NEI VFQ-25) was translated into Italian^21^ and constituted the VFQ-25.

### Rehabilitation procedure

The patient is seated 60–80 cm from the monitor of the pattern stimulator. After carefully cleaning the skin with “nuprep” gel, electrodes are applied using “Ten20” conductive paste configured as in VEP test recording. The negative electrode (black) is applied according to the 10–20 system of electrodes placed on the FPz position, the positive electrode (red) is placed on the Oz position and the Earth electrode (green) is placed in the Cz position.

The patient is fitted with a corrective lens over the selected eye (highest BCVA) for rehabilitation. The session is performed under scotopic conditions keeping the eye not undergoing training closed. Prior to initiating training, each patient is left to adapt to the ambient room lighting, until a natural pupil diameter of approximately 5 mm is obtained (about 10 min).

During the 10-min examination, a structured stimulus appears on the screen in the form of an alternating chess board at the reversal rate of 15 reversal/second with a fixation target (red point) in the center of the screen. In our study, we used the standard chessboard used in pattern 60 ‘VEP at the reversal rate of 15 reversal/s.

Whilst the patient fixes the target, a stimulus from the optical pathways through to the cortical visual areas is created and read by surface electrodes, which then produce a bioelectric VEP. During the session, the patient is instructed to maintain the target using the retinal area that produces the real-time sound (highest amplitude VEP with lower latency; a continuous, uninterrupted sound) corresponding to a better visual perception. The sound is more intense and continuous when the patient fixes the fixation target with a healthy area of the retina or becomes intermittent when the fixation is interrupted. The aim of the training is to ensure that the patient uses a retinal area with optimal biological activity. During the training sessions, operators (SP, VR) monitor the progress of the target fixation and the intensity of the sound, encouraging patients’ consistent attention to the sound produced. All examinations were performed in the same room, with the same lighting settings.

A complete ophthalmic examination was repeated for all patients at six weeks from baseline (T1). Clinical examination controlled for any ocular events over the study period which could have affected study results. Retinal sensitivity was retested at the same points, using the “retest mode.” This modality stores information about the landmarks used in previous exams, enabling a perfect overlapping of the new exam image with previous ones.

Statistical analysis was performed using the R software (A language and environment for statistical computing, version 3.3.3, R Foundation for Statistical Computing, Vienna, Austria). Given the non-normal distribution verified with the Shapiro–Wilk test and the small sample size, the pre- and post-rehabilitation values of each evaluated metric were compared with the nonparametric Wilcoxon test. Comparisons of paired samples (T0, T1) were performed with a one-sided Wilcoxon signed rank test and comparisons among treated and control samples were assessed with a Wilcoxon rank sum test (two-sided for pre-treatment and one-sided for post treated samples). Whiskers in box plots extend to the most extreme data point, which is no more than 1.5 times the interquartile range from the box. P-values were not corrected for multiple comparisons given the small number of comparisons. Differences were considered significant at *p* < 0.05.

## Results

Twenty-four patients (11 males and 13 females, 11 right eyes and 13 left eyes) with advanced AMD were included in the study. Fifteen patients were available for training and were enrolled into the training group and 9 patients were unavailable for training and were enrolled into the control group. Average patient age was 79.38 ± 6.94 (range 67–89 years). Patient demographic details are summarized in Table 1. No significant differences were observed between the groups for all considered parameters, reported in Tables 2 and 3.

**Table 1:** Table of subjects and fixation results in both groups.

Patient	Age (years)	Eye	Gender	Stability of fixation (% within 2° and 4°)	
RETIMAX				T0	T1
R1	75	R	F	Rel. Unst. 71%, 99%	Stable 83%, 100%
R2	72	R	M	Unstable 64%, 73%	Stable 97%, 99%
R3	68	R	M	Unstable 47%, 69%	Stable 80%, 93%
R4	87	L	M	Unstable 32%, 74%	Rel. Unst. 55%,100%
R5	89	L	F	Rel Unst 70%, 86%	Rel. Unst. 62%, 93%
R6	84	R	M	Rel. Unst. 53%, 98%	Rel. Unst. 71%,99%
R7	85	R	M	Rel. Unst. 66%, 85%	Rel Unst. 74%, 91%
R8	80	R	M	Rel. Unst. 74%, 96%	Rel Unst. 68%, 94%
R9	74	R	M	Rel Unst. 43%, 80%	Rel Unst. 65%, 95%
R10	89	L	F	Rel. Unst. 68%, 98%	Stable 83%,100%
R11	75	L	M	Rel. Unst. 70%, 78%	Rel. Unst. 70%, 94%
R12	88	L	F	Rel. Unst. 50%, 89%	Rel. Unst. 68%,100%
R13	67	R	M	Rel Unst . 58%, 91%	Stable 87%,100%
R14	79	R	F	Rel Unst. 39% 81%	Rel. Unst. 55%, 96%
R15	80	R	F	Rel Unst. 66%, 92%	Stable 78%, 91%
*Controls*					
C1	83	R	F	Rel. Unst. 72%, 99%	Rel. Unst. 72%, 95%
C2	83	R	F	Rel. Unst. 66%, 100%	Rel. Unst. 72%,99%
C3	84	L	F	Rel. Unst. 68%, 99%	Rel. Unst. 69%, 96%
C4	80	R	F	Rel. Unst. 37%, 98%	Rel. Unst. 42% 96%
C5	71	L	M	Rel. Unst. 42%, 95%	Rel. Unst. 55% 91%
C6	87	L	F	Rel. Unst. 73%,100%	Unstable 57%, 74%
C7	78	L	M	Rel. Unst. 70%,99%	Rel. Unst. 74%,100%
C8	67	R	F	Rel. Unst. 66%,99%	Rel. Unst. 59%,93%
C9	80	R	F	Rel. Unst. 72%,99%	Rel. Unst. 55%,98%

*R* right eye, *L* left eye, *Rel.Unst.* relatively unstable; *T0* baseline; *T1* 6-week follow-up, *RETIMAX* patients who completed the training sessions, *CONTROLS* patients unavailable to attend the training sessions.

**Table 2:** Table of results in both groups.

	RETIMAX (15)			Controls (9)		
	T0	T1	*p* value	T0	T1	*p* value
BCVA (*ETDRS*)	20.33	27.07	< 0.001	20.67	18.56	0.99
Contrast sensibility (*logSC*)	1.17	1.31	0.042	1.23	1.17	0.97
Reading acuity (*logMar*)	0.29	0.27	0.13	0.33	0.34	0.98
Reading speed (*WpM*)	31.60	33.91	0.19	43.82	29.07	1.00
Quality of life (*VFQ-25*)	48.50	54.29	< 0.001	41.86	37.31	0.99
Macular sensitivity (*dB*)	5.79	5.95	0.40	6.17	6.06	0.53
Fixation 2° (*%*)	61.07	69.73	0.13	68	65.22	0.59
Fixation 4° (*%*)	89.13	95.53	0.019	98.33	96.11	0.61

T0 = baseline values; T1 = values at 6-week follow-up.

**Table 3:** Comparison of the results of the two groups (%) for the considered parameters with statistical significance.

	Retimax (%)	Controls (%)	*p* value
BCVA	+ 33.12	− 10.22	0.03
Contrast sensitivity	+ 11.97	− 5.41	0.07
Reading acuity	+ 9.09	− 3.33	0.90
Reading speed	+ 7.29	− 33.66	0.08
QoL	+ 11.93	− 10.88	0.001
Retinal sensibility	+ 2.76	− 1.80	0.51
Fixation 2°	+ 13.06	− 4.09	0.79
Fixation 4°	+ 7.18	− 2.26	0.82

**Figure 1:**
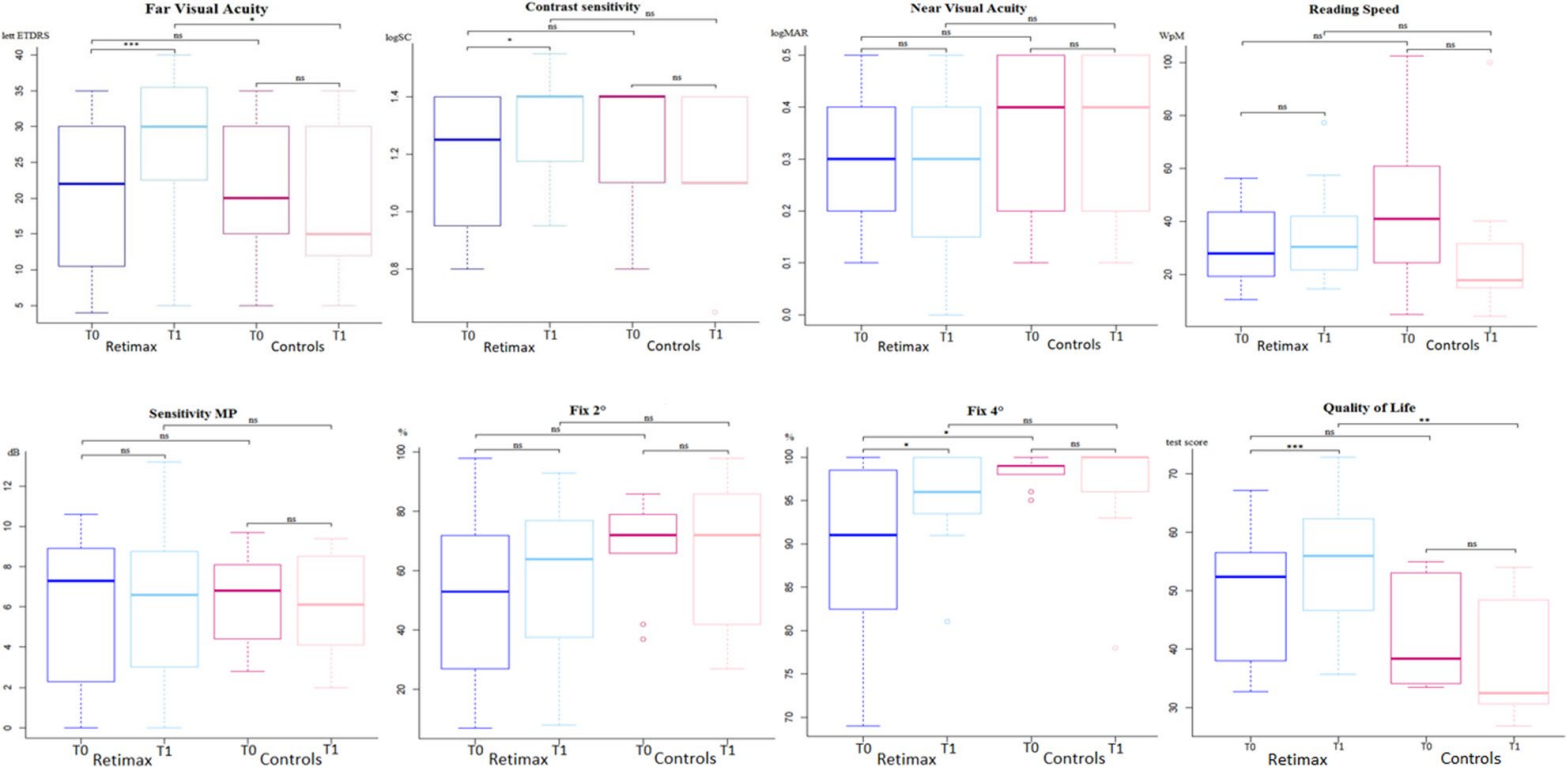
Box plot for baseline (T0) and 6 weeks (T1) assessments in the two groups. The horizontal bar of the box represents the median. ns (*p* > 0.05); *(*p* < .05); **(*p* < .01); ***(*p* < .001).

**Figure 2:**
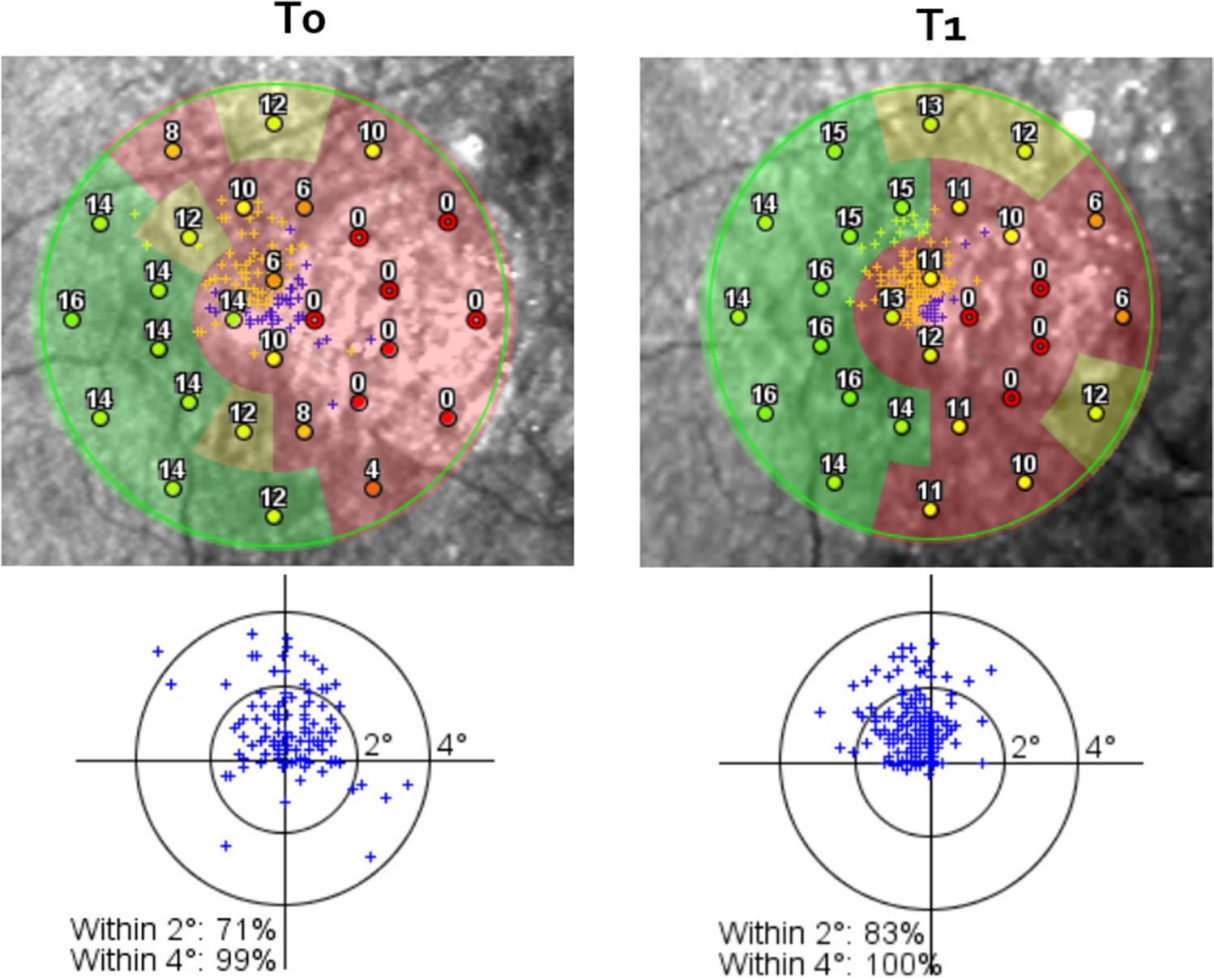
Microperimetry and fixation stability at baseline and after the training sessions in patient R1.

**Figure 3:**
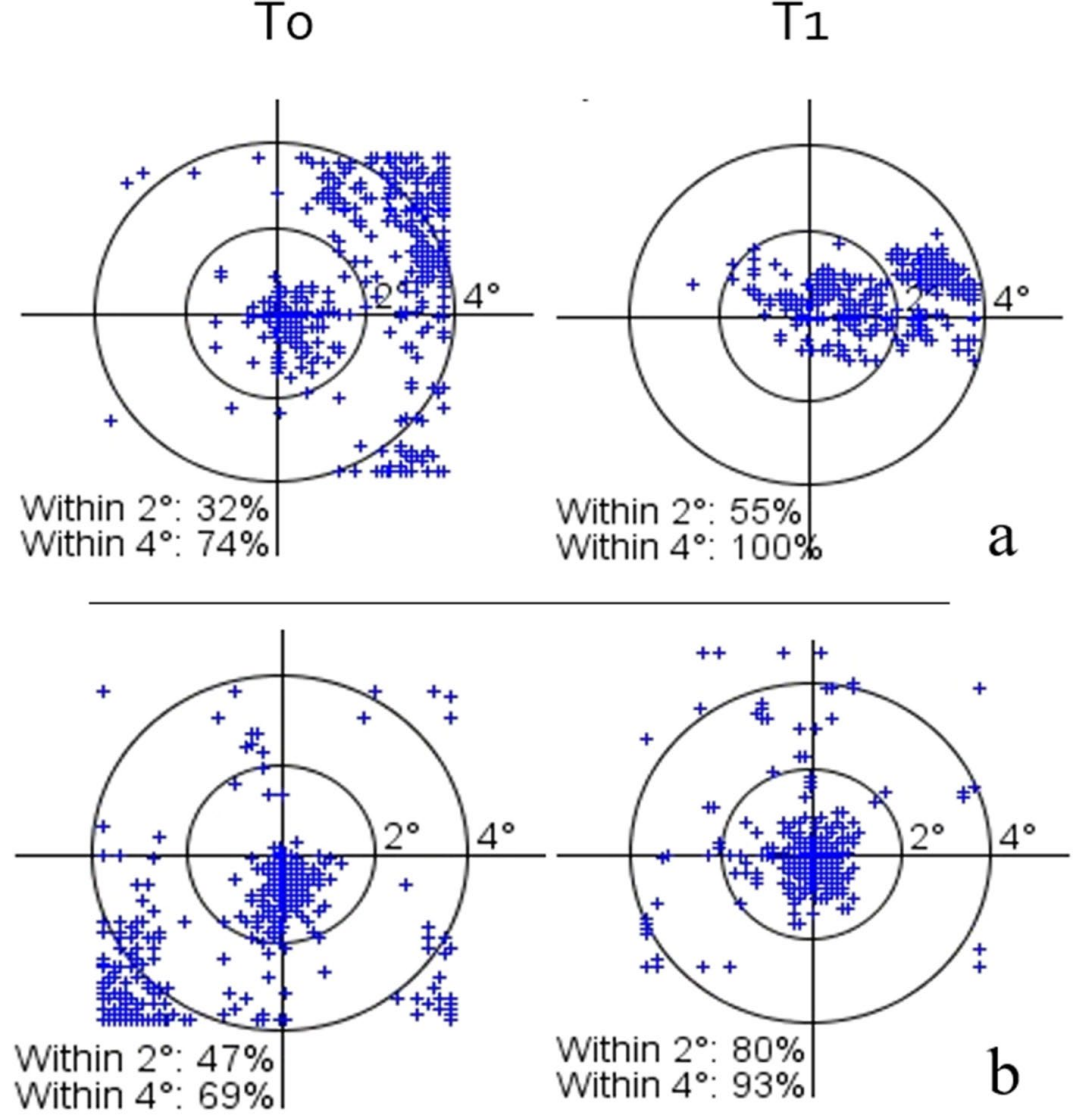
Fixation stability at baseline (T0) and after 6 weeks(T1) in patients R4 (**a**) and R3 (**b**).

In the Retimax group, BCVA, CS, quality of life and 4° fixation stability improved significantly. Insignificant improvements were also observed in reading ability, macular sensitivity and 2° fixation acuity (Table 2; Figs. 1, 2). Patients’ visual fixation passed from relatively unstable to stable in 6 (40%) in the Retimax group (Table 1; Fig. 3), whereas all parameters either remained unchanged or worsened in the control group (Table 2).

At T6, a mean BCVA improvement of 33% was observed for patients in the Retimax group compared to a mean worsening of 10% reported in the control group (*p* = 0.03; Table 3). Similar changes were observed for quality of life, where a mean improvement of + 12% was recorded for patients in the Retimax Group vs. 11% in the control group (*p* = 0.02; Table 3).

## Discussion

Our study suggests that VEP auditory biofeedback rehabilitation is a useful method to improve residual visual function in patients with advanced AMD. As has been proven for amblyopia^18^ and acquired cerebral damage^19^, VEP training seems to stimulate a re-adaptation of the vision pathway, thereby significantly improving visual acuity following rehabilitation, also in patients with low vision for advanced AMD.

The results of our investigation show an improvement of BCVA, CS, fixation stability and quality of life in comparison to a control group. Our data are in agreement with previous studies that showed stabilization of fixation and consequent improvement in visual acuity, retinal sensitivity and reading abilities in patients with AMD and different macular diseases using a microperimetric biofeedback training^22–26^.

Differently from microperimetric training, VEP visual rehabilitation is based on the training of patient’s spontaneous PRL by guiding the patient through an independent search for fixation on the retinal point that generates a wider VEP response, inducing a progressive voluntary control of eye movements. The process is therefore operator-independent. Microperimetric rehabilitation is based on the training of a PRL established by the physician and auditory biofeedback is therefore generated according to the proximity of patients’ fixation to this predefined healthy area of the retina. Results reported for Microperimetric rehabilitation for AMD patients have been few, but promising. A small study of 5 AMD patients treated with microperimeter (Nidek MP-1) biofeedback training (10 sessions/10 min) was reported by Vingolo et al.^22^. The authors reported significant improvements in all parameters considered. A larger study of 15 AMD patients with the same device (Nidek MP-1) reported increased fixation stability with improved reading abilities^15^.

Optimization with specific training was demonstrated by Tarita-Nistor et al.^14^ in the exploration of plasticity of fixation. The PRL was relocated in 6 AMD patients with the assistance of auditory biofeedback (Nidek MP-1) and fixation and reading speed were improved by 53% and 38% respectively, following training.

In a recent meta-analysis^23^, including 9 studies (885 eyes), reading speed was reported to be significantly improved (regardless of rehabilitation type) in AMD patients treated with microperimeter biofeedback rehabilitation systems, whilst mood and depression scores remained unchanged.

Two recent articles^24,25^ compared the effectiveness of two different techniques of low vision rehabilitation with the MP-1 microperimeter in AMD patients. The techniques included a standard sound biofeedback or a luminous flickering biofeedback stimulus in place of the fixation target. Vingolo et al. ^24^ showed that the biofeedback method associated with the projection of a visual luminous pattern presented better results in training the patients to modify their PRL. Amore et al.^25^ noted more benefits associated with the flickering pattern biofeedback training compared to standard feedback for all visual function assessments considered.

The MAIA microperimeter has also be used for biofeedback rehabilitation. A recent study confirmed its utility in healthy retinal area training of 77 consecutive AMD patients with bilateral central vision loss^17^. Daibert-Nido et al.^26^, in a retrospective review of 30 cases, also reported improvements for visual acuity, however with a negative trend for fixation stability, confirming the utility of the same tool in AMD patients.

In our study, a control group who did not receive training was included. Complete ophthalmic examinations were performed during the same study period under the same clinical settings. As expected, we found improvements for all parameters considered in the Retimax group, especially for BCVA, CS, quality of life and 4° fixation stability over the study period. In the control group, all parameters either remained unchanged or worsened. Comparisons between the groups highlighted an improvement in favour to the Retimax group, especially for BCVA and quality of life.

The majority of the patients in the Retimax group reported subjective improvements in everyday skills and a better confidence in fixation tasks. Most patients requested the continuation of rehabilitation sessions. We speculate that further rehabilitative sessions may be required to maintain the achieved improvements and may further improve results.

We are aware of some limitations of our study. Firstly, microperimetric sensitivity and fixation values may be inaccurate due to the unstable fixation of patients despite the eye-tracking system present in the OCT machine. Moreover, reading data could be inaccurate based on many possible extrinsic variabilities (eg. patient fatigue or concern). Finally, a larger sample size, patient randomization and longer follow-up are needed to investigate the duration of improved efficacy over time.

## Conclusions

VEP rehabilitation with Retimax low-vision training system optimizes residual vision function in AMD patients. Fixation is stabilized with improved visual acuity, contrast sensitivity and quality of life. Our study confirms the evidence of the plasticity of the visual system that can be successfully trained to optimize residual vision, creating new connections between the retina and the visual cortex with a rearrangement of neurons in this area. Further studies are needed to confirm these results and determine the duration of treatment over time.
